# Incidence of Pneumothorax and Pneumomediastinum in 497 COVID-19 Patients with Moderate–Severe ARDS over a Year of the Pandemic: An Observational Study in an Italian Third Level COVID-19 Hospital

**DOI:** 10.3390/jcm10235608

**Published:** 2021-11-29

**Authors:** Nardi Tetaj, Gabriele Garotto, Fabrizio Albarello, Annelisa Mastrobattista, Micaela Maritti, Giulia Valeria Stazi, Maria Cristina Marini, Ilaria Caravella, Manuela Macchione, Giada De Angelis, Donatella Busso, Rachele Di Lorenzo, Silvana Scarcia, Anna Farina, Daniele Centanni, Joel Vargas, Martina Savino, Alessandro Carucci, Andrea Antinori, Fabrizio Palmieri, Gianpiero D’Offizi, Stefania Ianniello, Fabrizio Taglietti, Paolo Campioni, Francesco Vaia, Emanuele Nicastri, Enrico Girardi, Luisa Marchioni, ICU COVID-19 Study Group

**Affiliations:** 1 UOC Resuscitation, Intensive and Sub-Intensive Care, National Institute for Infectious Diseases IRCCS, Lazzaro Spallanzani, 00149 Rome, Italy; gabriele.garotto@inmi.it (G.G.); micaela.maritti@inmi.it (M.M.); giuliavaleria.stazi@inmi.it (G.V.S.); mariacristina.marini@inmi.it (M.C.M.); ilaria.caravella@inmi.it (I.C.); manuela.macchione@inmi.it (M.M.); giada.deangelis@inmi.it (G.D.A.); donatella.busso@inmi.it (D.B.); rachele.dilorenzo@inmi.it (R.D.L.); silvana.scarciadaprano@inmi.it (S.S.); anna.farina@inmi.it (A.F.); alessandro.carucci@inmi.it (A.C.); luisa.marchioni@inmi.it (L.M.); 2 Department of Radiology and Diagnostic Imaging, National Institute for Infectious Diseases IRCCS Lazzaro Spallanzani, 00149 Rome, Italy; fabrizio.albarello@inmi.it (F.A.); stefania.ianniello@inmi.it (S.I.); paolo.campioni@inmi.it (P.C.); 3 Respiratory Infectious Diseases Unit, National Institute for Infectious Diseases IRCCS Lazzaro Spallanzani, 00149 Rome, Italy; a.mastrobattista@inmi.it (A.M.); fabrizio.palmieri@inmi.it (F.P.); 4 Clinical and Research Department of Infectious Diseases, National Institute for Infectious Diseases IRCCS Lazzaro Spallanzani, 00149 Rome, Italy; daniele.centanni@inmi.it (D.C.); andrea.antinori@inmi.it (A.A.); gianpiero.doffizi@inmi.it (G.D.); fabrizio.taglietti@inmi.it (F.T.); emanuele.nicastri@inmi.it (E.N.); 5 Department of Anesthesiology, Intensive Care and Emergency Medicine, Fondazione Policlinico Universitario A. Gemelli IRCCS, 00168 Rome, Italy; joel.vargas@policlinicogemelli.it (J.V.); martina.savino@policlinicogemelli.it (M.S.); 6 Health Direction, National Institute for Infectious Diseases IRCCS Lazzaro Spallanzani, 00149 Rome, Italy; francesco.vaia@inmi.it; 7 Scientific Direction, National Institute for Infectious Diseases IRCCS Lazzaro Spallanzani, 00149 Rome, Italy; enrico.girardi@inmi.it

**Keywords:** COVID-19, barotrauma, pneumothorax, pneumomediastinum, subcutaneous emphysema, intensive care unit, non-invasive ventilation, invasive mechanical ventilation, COVID-19 waves

## Abstract

(1) Background: COVID-19 is a novel cause of acute respiratory distress syndrome (ARDS). Indeed, with the increase of ARDS cases due to the COVID-19 pandemic, there has also been an increase in the incidence of cases with pneumothorax (PNX) and pneumomediastinum (PNM). However, the incidence and the predictors of PNX/PMN in these patients are currently unclear and even conflicting. (2) Methods: The present observational study analyzed the incidence of barotrauma (PNX/PNM) in COVID-19 patients with moderate–severe ARDS hospitalized in a year of the pandemic, also focusing on the three waves occurring during the year, and treated with positive-pressure ventilation (PPV). We collected demographic and clinical data. (3) Results: During this period, 40 patients developed PNX/PNM. The overall incidence of barotrauma in all COVID-19 patients hospitalized in a year was 1.6%, and in those with moderate–severe ARDS in PPV was 7.2% and 3.8 events per 1000 positive-pressure ventilator days. The incidence of barotrauma in moderate–severe ARDS COVID-19 patients during the three waves was 7.8%, 7.4%, and 8.7%, respectively. Treatment with noninvasive respiratory support alone was associated with an incidence of barotrauma of 9.1% and 2.6 events per 1000 noninvasive ventilator days, of which 95% were admitted to the ICU after the event, due to a worsening of respiratory parameters. The incidence of barotrauma of ICU COVID-19 patients in invasive ventilation over a year was 5.8% and 2.7 events per 1000 invasive ventilator days. There was no significant difference in demographics and clinical features between the barotrauma and non-barotrauma group. The mortality was higher in the barotrauma group (17 patients died, 47.2%) than in the non-barotrauma group (170 patients died, 37%), although this difference was not statistically significant (*p* = 0.429). (4) Conclusions: The incidence of PNX/PNM in moderate–severe ARDS COVID-19 patients did not differ significantly between the three waves over a year, and does not appear to be very different from that in ARDS patients in the pre-COVID era. The barotrauma does not appear to significantly increase mortality in COVID-19 patients with moderate–severe ARDS if protective ventilation strategies are applied. Attention should be paid to the risk of barotrauma in COVID-19 patients in noninvasive ventilation because the event increases the probability of admission to the intensive care unit (ICU) and intubation.

## 1. Introduction

Coronavirus disease 2019 (COVID-19) is an infectious disease caused by a coronavirus discovered in December 2019, named severe acute respiratory syndrome coronavirus-2 (SARS-CoV-2), and it is responsible for the current global pandemic that started in March 2020 [1].

SARS-CoV-2 is transmitted from person-to-person via droplets, contact, and from aerosolized particles [2].

COVID-19 patients with progressive severe disease should be monitored closely for worsening respiratory status; some patients may progress to acute respiratory distress syndrome (ARDS), and typically require an incremental respiratory support [3]. Noninvasive positive-pressure ventilation (NIPPV) may be beneficial in avoiding intubation in moderate–severe ARDS COVID-19, especially under conditions of resource constraints or to avoid overwhelming critical care resources. Orotracheal intubation and invasive positive-pressure ventilation (IPPV) are required for patients with persistent hypoxemia.

COVID-19 is an important novel cause of ARDS [4]. The higher incidence of barotrauma in ARDS patients has been reported as early as the pre-COVID era in several studies [5,6].

Indeed, with the increase of ARDS cases due to the COVID-19 pandemic, there has also been an increase in the incidence of cases with pneumothorax (PNX) and pneumomediastinum (PNM) [7,8,9,10].

CT imaging can demonstrate typical patterns of imaging manifestations that could be used to diagnose COVID-19. The most common distribution patterns are bilateral, peripheral/subpleural posterior consolidations, ground glass opacities (whether in isolation or coexisting with other lesion-like consolidations), interlobular septal thickening, and crazy paving [11].

However, the incidence, the pathogenesis, and the predictors of PNX/PMN in COVID-19 patients are currently unclear and even conflicting [12,13].

The aim of this observational single center study is to assess the following:

- The incidence of PNX/PNM in COVID-19 patients over a year, as the number of patients with PNX/PNM from COVID-19 patients, and as the number of barotrauma events during 1000 days of non-invasive or invasive positive-pressure ventilation;
- The incidence between the three waves of COVID-19 during a year of the pandemic;
- The predictive factors associated with the development of PNX/PNM in COVID-19 patients.

## 2. Materials and Methods

### 2.1. Study Design and Participants

The study was conducted at the National Institute for Infectious Disease (INMI) “Lazzaro Spallanzani” in Rome, Italy, which is a third level COVID-19 center with a 200-bed hospital for infectious diseases and a 50-bed ICU. This observational cohort study included adult COVID-19 patients hospitalized in our COVID-center from 1 April 2020 to 1 April 2021. 

To simplify the reading of the article we have assigned the common term “barotrauma”, referring to the manifestations of two pathological states: pneumothorax (PNX) and pneumomediastinum (PNM).

The inclusion criteria were adult patients with confirmed COVID-19 by nasal pharyngeal swab for reverse transcriptase polymerase chain reaction (rtPCR) assay, hospitalized to our hospital, with a diagnosis of moderate–severe ARDS COVID-19 and the presence of either pneumothorax or pneumomediastinum.

Patients with subcutaneous emphysema (SE) alone were not included in the barotrauma group. During the data collection we included the latter in the study; however, since we only had three patients with subcutaneous emphysema alone, and no air was found in the pleura or mediastinum when they underwent further radiological investigations with chest CT, in the end they were excluded from the study.

All patients underwent a chest CT scan at admission and subsequent CT scans when ventilatory parameters exacerbated or to monitor disease progression.

Pneumothorax (PNX) is defined by the presence of air in the pleural cavity with or without collapse of the lung, while pneumomediastinum (PNM) is the presence of extraluminal gas within the mediastinum and subcutaneous emphysema (SE), the infiltration of air in the subcutaneous layer of the skin [14].

Moderate–severe ARDS COVID-19 was reported on thoracic CT scans and met Berlin criteria [15], where disease severity included a PaO_2_/FiO_2_ ratio < 200 mmHg with a positive end-expiratory pressure (PEEP) ≥ 5 cm H_2_O [4]. The patients with diagnosed moderate–severe ARDS were treated with positive-pressure ventilation (PPV), including noninvasive positive-pressure ventilation (NIPPV) and invasive positive-pressure ventilation (IPPV). The NIPPV included continuous positive airway pressure (CPAP) via a helmet, Boussignac mask, or ventilation machine, and bilevel positive airway pressure (BiPAP) via a ventilator machine. Patients were managed according to recommendations from published guidelines on protective ventilation, which is an optimal PEEP with a target peak inspiratory pressure (PIP) less than 30 cm H_2_O, tidal volume from 6 to 8 mL/kg of ideal body weight (IBW), plateau pressure ≤ 30 cm H_2_O, and driving pressure ≤ 15 cm H_2_O, to keep the risk of barotrauma low [16,17].

### 2.2. Data Collection

The data collected included age, gender, body mass index (BMI), sequential organ failure assessment score (SOFA) at hospital admission, acute physiology and chronic health evaluation II score (APACHE II) at hospital admission, previous hospitalization in the last 6 months, previous surgical procedures in the last month, days in PPV pre-PNX/PNM and pre-orotracheal intubation (OTI), and length of hospital stay.

The comorbidities included arterial hypertension, other heart diseases, diabetes, kidney disease (stage 3–5 of chronic kidney disease, CKD), moderate to severe liver disease, chronic pulmonary diseases (which included chronic obstructive pulmonary disease COPD, bronchial asthma, emphysema bullae, or other pulmonary diseases), neoplasm during the last 5 years (which included solid neoplasia or hematological malignancy), chronic neurological disorders, autoimmune diseases, obesity (defined as BMI > 30 kg/m^2^), and other diseases. Cohort data were collected prospectively following specific criteria and definitions, through the institutional electronic health record and Microsoft Access. All patients gave informed consent for the collection of personal data for research purposes.

### 2.3. Statistical Analysis

Quantitative variables were tested for normal distribution and compared by means of a two tailed t-test. Differences in group proportions were assessed using the X^2^ test and Fisher’s exact test. Continuous variables are expressed as median (interquartile range, IQR) or mean (± standard deviation, SD) with a 95% confidence interval (95% CI) and compared with the Pearson test. Nominal data are expressed as N (percentages, %). The abovementioned cohorts were analyzed by survival analysis. Two-tailed *p*-values < 0.05 were considered statistically significant. The relative incidence of PNX/PNM was expressed as events per 1000 days or percentages (%) in positive-pressure ventilation COVID-19 patients. Statistical analysis was performed through the combination of STATA (Stata Corp LP, College Station, TX, USA) and SPSS 27 (IBM Corp, Armonk, NY, USA) software.

## 3. Results

**Figure 1:**
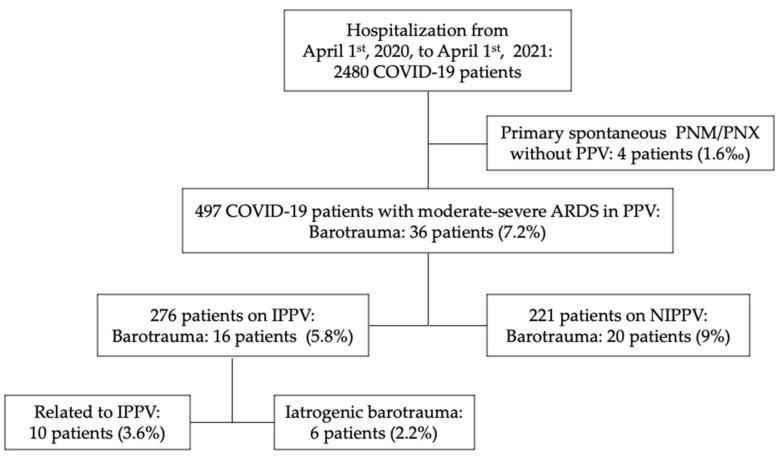
Flowchart of study selection. Abbreviations: COVID-19, coronavirus 2019; PPV, positive-pressure ventilation; NIPPV, noninvasive positive-pressure ventilation; IPPV, invasive positive-pressure ventilation; barotrauma is referred to pneumothorax/pneumomediastinum.

From 1 April 2020 to 1 April 2021, a total of 2480 COVID-19 patients were admitted to our COVID hospital. Of these, 497 (20%) with moderate–severe ARDS COVID were treated with PPV, of which 276 were on IPPV (11%) and 221 were on NIPPV (9%), Figure 1.

Forty patients developed PNX/PNM and three patients developed isolated subcutaneous emphysema (SE); nonetheless, we did not include the latter in the barotrauma category.

Four patients had PNX/PNM at the beginning of the hospital stay with no supplemental positive-pressure oxygen treatment’ we included them as the primary spontaneous barotrauma group, and their incidence over a year was 0.16% (4 cases/2480 admitted COVID-19 patients).

**Figure 2:**
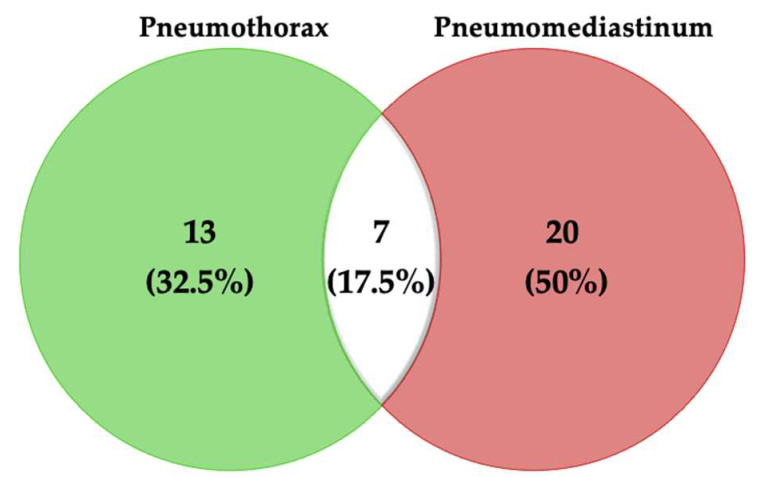
Venn diagram showing the patients with different clinical manifestations of barotrauma.

The Venn diagram in Figure 2 shows 13 patients with isolated PNX (32.5%), 20 of them with PNM (50%), and 7 patients with both PNM and PNX (17.5%).

**Figure 3:**
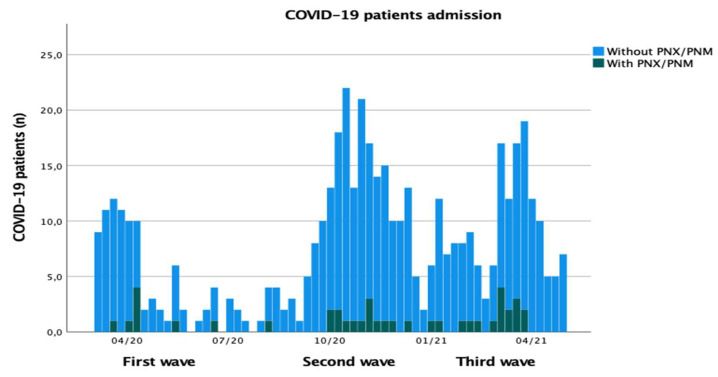
Histogram of weekly incidence of hospital admission of COVID-19 patients and PNX/PNM diagnosis over a year by pandemic waves. Legend: PNX, pneumothorax; PNM, pneumomediastinum; COVID-19, coronavirus disease 2019; light blue, COVID-19 patients; dark grey, PNX/PNM diagnosis; n, absolute numbers.

We took 31 August 2020 as the boundary between the first and second wave of the pandemic, and 1 January 2021 as the point of demarcation between the second and third wave (Figure 3).

### 3.1. Incidence of Barotrauma in All COVID-19 Patients Admitted during a Year

The overall incidence of barotrauma (including non-PPV, primary spontaneous barotrauma group, 4 patients) in all COVID-19 patients, with or without ARDS, admitted to the hospital during the first wave was 1.43% (9 cases in 627 COVID-19 patients), in the second wave was 1.45% (15 cases in 1037 COVID-19 patients), and in the third wave was 1.96% (16 cases in 816 COVID-19 patients). 

**Figure 4:**
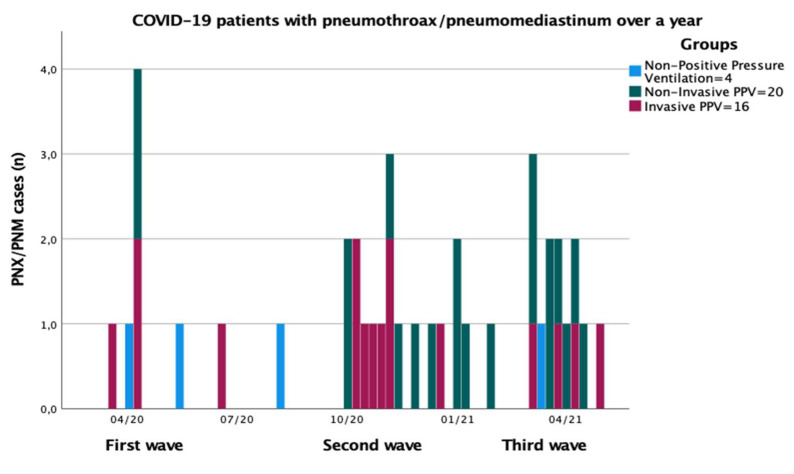
Histogram of weekly incidence of PNX/PNM diagnosis in COVID-19 patients over a year by pandemic waves. Legend: PNX, pneumothorax; PNM, pneumomediastinum; COVID-19, coronavirus disease 2019; PPV, positive-pressure ventilation; light blue, patients with non-PPV; dark grey, patients with non-invasive PPV; red, patients in invasive PPV; *n*, absolute numbers.

The overall incidence of barotrauma in all hospitalized COVID-19 patients over a year was 1.6% (40 cases in 2480 COVID-19 patients) (Figure 3 and Figure 4).

### 3.2. Incidence of Barotrauma in COVID-19 Patients Treated with PPV

The incidence of barotrauma in COVID-19 patients treated with PPV (which is meant as NIPPV+IPPV) during the first wave was 7.8% (7 cases/115 PPV pts), during the second wave was 7.4% (14 cases/201 PPV pts), and during the third wave was 8.7% (15 cases/181 PPV pts).

The incidence of barotrauma over a year in COVID-19 patients treated with PPV was 7.2% (36 cases/497 PPV pts) with 3.8 events per 1000 positive-pressure ventilator days.

### 3.3. Incidence of Barotrauma Due to Non-Invasive PPV

Twenty COVID-19 patients in non-invasive PPV (NIPPV) presented barotrauma, of which 2 patients had only pneumothorax, 11 patients had pneumomediastinum, and 7 patients had PNM in addition to PNX.

Treatment with noninvasive positive-pressure respiratory support alone was associated with an incidence of PNX/PNM of 9.1% (20 cases/219 moderate–severe ARDS COVID-19 patients in NIPPV) and 2.6 events per 1000 non-invasive ventilator days (20 cases per 3503 non-invasive ventilator days).

During the hospital stay prior to PNX/PNM, 16 subjects used BiPAP ventilation and 4 subjects used CPAP ventilation.

All 20 patients were hospitalized in the non-critical care ward at the barotrauma event, and 19 patients (95%) were admitted to the ICU after the event, of which 16 (80%) subsequently underwent orotracheal intubation (OTI) due to a worsening of respiratory parameters.

### 3.4. Incidence of Barotrauma Due to Invasive PPV

Sixteen patients of the barotrauma group had PNM/PNX during invasive mechanical ventilation, of which 9 presented with pneumothorax and 7 with pneumomediastinum. All patients with PNX needed chest drainage and only one with PNM needed mediastinal drainage. The incidence of barotrauma in ICU COVID-19 patients on invasive PPV over a year was 5.8% (16 cases per 276 pts in IPPV) and 2.7 events per 1000 invasive ventilator days (16 cases per 5996 invasive ventilator days).

A total of 3.6% (10/276) of cases were related to mechanical ventilation and 2.2% (6/276) were related to iatrogenic barotrauma.

Six patients had barotrauma due to an iatrogenic cause, attributable to diagnostic or therapeutic procedures such as thoracentesis (2 patients, 12.5%), bronchoalveolar lavage (BAL) through a fiberoptic bronchoscope (2 patients, of which 1 had tracheomalacia and a tracheoesophageal fistula, 12.5%), insertion of a central venous catheter through subclavian vein access (1 patient, 6%), and the placement of a nasogastric tube (1 patient with tracheomalacia, 6%).

### 3.5. Barotrauma versus Non-Barotrauma Group

In regard to demographic and clinical features, no significant difference was observed between the PNX/PNM and non-PNX/PNM group, both representing the 497 COVID-19 patients with moderate–severe ARDS who underwent PPV, which included NIPPV and IPPV, Table 1.

The median age of this cohort was 61.5 years (IQR, 55.7–71.5); of those, 29 were males (82.5%) and most were overweight with a median body mass index (BMI) of 26.9 kg/m^2^ (IQR, 26–29.4). The most frequent comorbidities were hypertension (64%), obesity (30.5%), other cardiopathies (19.4%), chronic pulmonary disease (16.7%), and diabetes (13.8%). Comorbidities and demographic features were compared between the PNX/PNM and non-PNX/PNM group, and no significant differences were found. Chronic pulmonary disease was not associated with PNX/PNM (*p* = 0.582).

Four patients (11%) were diagnosed incidentally, the others were associated with marked respiratory deterioration, and 21 patients (58.3%) were associated with subcutaneous emphysema. In 6 patients, the barotrauma was suspected with a chest ultrasound exam, performed after a worsening of the ventilator parameters, and then confirmed with a chest CT scan.

The mortality appeared to be higher in the PNX/PNM group (17 patients died, 47.2%) than in the non-PNX/PNM group (170 patients died, 37%), although this difference was not statistically significant (*p* = 0.429).

## 4. Discussion

The incidence of PNX/PNM in moderate–severe ARDS COVID-19 patients did not differ significantly between the three waves (7.8%, 7.4%, and 8.7%, respectively), which does not appear to be very different from that in ARDS patients in the pre-COVID era, which was below 10%, using lung-protective ventilation strategies [16,17].

**Figure 5:**
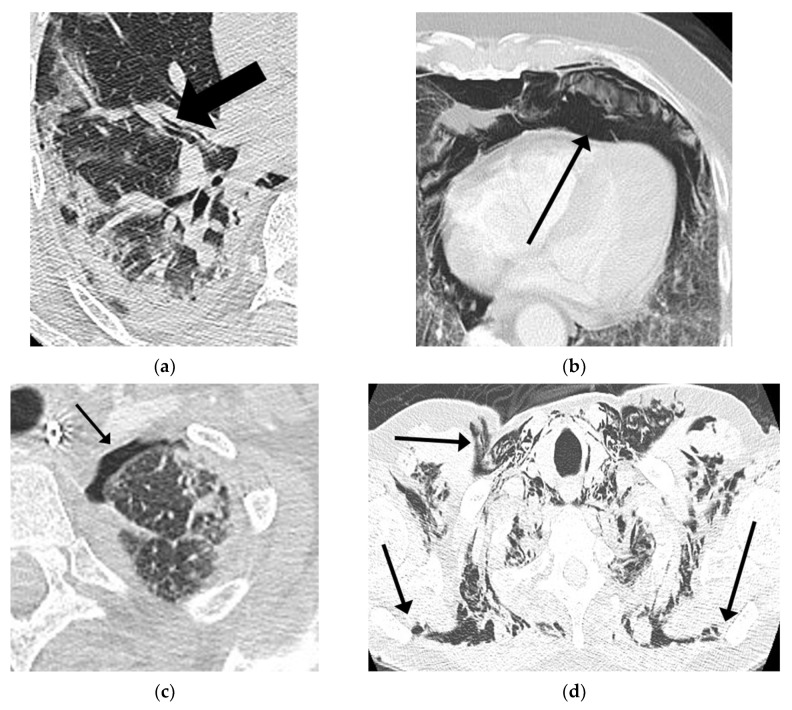
Chest CT in patients of our study with ARDS COVID-19 pneumoniae showing: (**a**) Macklin effect; (**b**) pneumomediastinum; (**c**) pneumothorax; and (**d**) subcutaneous emphysema.

Chest ultrasound is an easy-to-use bedside tool in COVID-19 patients, which can make an important contribution when patients experience respiratory exacerbation. The BLUE-protocol was applied during bedside thoracic ultrasound exams, which investigated the absence of lung sliding and B-lines, the presence of the stratosphere sign (pleural line with A-lines), or the lung point [18]. In the presence of these signs, a chest CT scan was performed to evaluate the extension of the barotrauma. Also, all patients requiring prolonged positive-pressure ventilation underwent repeated chest imaging to monitor the disease progression (Figure 5).

Histologic examinations indicated diffuse alveolar damage (DAD) due to the excessive and prolonged production of inflammatory cytokines, known as a cytokine storm, with interstitial/alveolar lymphocytic infiltration and a paucity of neutrophils. Furthermore, microvascular injury and thrombosis were regularly found within small lung capillaries and inflammatory perivascular aggregates in the septa and alveolar space. In addition, an organizing pneumoniae with fibrosis and type II pneumocyte hyperplasia can be observed following the early stages of the disease [19]. Also, there is general agreement that the direct viral infection of type 1 and type 2 pneumocytes makes alveoli more liable to rupture, resulting in alveolar membrane rupture [20]. The Macklin effect is described as the rupture of the alveolar tree associated with increased intra-alveolar pressure that could release air that subsequently dissects the peri-bronchovascular sheath [21,22]. These alveolar ruptures are either isolated or confluent and then result in pulmonary lacerations. This radiologic appearance could be identified on the chest CT, representing macroscopic evidence of the proposed virus-induced frailty of airways tissue (Figure 5a). It is possible that the coexistence of all these factors can lead to the increased risk of developing PNX/PNM, especially in patients treated with prolonged positive-pressure ventilation [23,24].

The incidence of primary spontaneous barotrauma over a year in COVID-19 patients without supplemental oxygen was 0.16%, more frequent than in the non-COVID-19 population [23]. All four patients were neglected at home and refused hospitalization even in the presence of dyspnea and a high fever, up to significant clinical aggravations such as chest pain, tachypnoea, and hypoxemia. Indeed, three of those already had moderate–severe ARDS and barotrauma upon entering the hospital.

The COVID-19 patients with moderate–severe ARDS in NIPPV had an incidence of 9.1% over a year and 2.6 events per 1000 non-invasive ventilator days. In awake patients, a prolonged increased inspiratory effort, hyperventilation, transpulmonary pressure swings, coughing fits, and non-adaptation to noninvasive positive-pressure ventilation could put them at risk of patient self-inflicted lung injury (P-SILI), reported in ARDS patients treated with NIPPV [25]. This could precipitate to an increase of intra-alveolar pressure, causing further damage to the lung and as a consequence a predisposition to barotrauma. The occurrence of barotrauma requires careful clinical monitoring and is associated with a high probability of ICU admission as well as a high probability of orotracheal intubation. Long-term NIPPV treatment, especially in the absence of clinical improvement, should consider the inclusion of repeating chest radiology imaging to assess the evolution of COVID-19 pneumonia and its potential complications.

The incidence of PNX/PNM in patients in mechanical ventilation was 5.8% over a year, of which 3.6% was related to MV and 2.2% was due to an iatrogenic cause attributable to diagnostic or therapeutic procedures. This incidence appears to be lower from that in COVID-19 patients treated with NIPPV (9.1%), applying lung-protective ventilation strategies and keeping with national guidelines for the management of ARDS [16]. Patients on invasive mechanical ventilation were controlled by sedation, analgesia, and curarization when necessary, so that they could be optimally adapted to protective mechanical ventilation by choosing an optimal driving pressure and plateau pressure (≤14 cm H_2_O and ≤30 cm H_2_O, when it was possible) in order to reduce the risk of barotrauma [26]. This may be the reason why we have had a lower incidence in intubated patients than that documented in some other recent studies [27,28]. Furthermore, the incidence of barotrauma due to iatrogenic causes in the ICU (2.2%), mostly from thoracentesis and fibrobronchoscopy, was no higher than that documented in several studies [29,30,31,32].

Comorbidities and demographic features were compared between the PNX/PNM and non-PNX/PNM group, and no significant differences were found. COPD, bronchial asthma, pulmonary emphysema bullae, or other chronic pulmonary diseases were not associated with the development of PNX/PNM in COVID-19 patients, which is consistent with several other studies [7,23], and this could be explained by the fact that all patients were managed by physicians according to ARDS published guideline recommendations as early as the pre-COVID era and good medical practice on protective ventilation, to keep the risk of barotrauma and self-inflicted lung injury low.

This study has several limitations. First, this is an observational study designed in a single third level COVID-19 center, and some patients were admitted from other hospitals already with barotrauma, which could somewhat overestimate the results presented in the current study. Second, controlling for confounders as in any observational study may be incomplete despite all efforts, and we did not adjust the incidence of barotrauma for all potential influencing confounders. Third, even though the study included one year of observation, the number of participating COVID-19 patients with PNX/PNM was low (40 patients), and the small sample size of the study cannot provide a reliable 95% confidence interval or precise estimates about predicted factors; for this reason, the results of this study need to be confirmed in further research with larger studies.

However, this study also has some strengths: observational studies are the first step to formulate a hypothesis that could incentivize the design of larger studies and the findings of this study seemed consistent with data already presented in medical literature. The study recorded COVID-19 patients during a year in a single third level COVID-center, which included three waves of the pandemic.

## 5. Conclusions

In summary, the incidence of PNX/PNM in moderate–severe ARDS COVID-19 patients did not differ significantly between the three waves over a year, and does not appear to be very different from barotrauma in ARDS patients in the pre-COVID era.

Clinicians should be aware of the risk of barotrauma in any COVID-19 patient with moderate–severe ARDS, especially when they are in NIPPV where the patient is awake and can experience self-inflicted lung injury. The occurrence of barotrauma is associated with a high probability of ICU admission and of OTI requirement.

PNX/PNM does not appear to significantly increase mortality in COVID-19 patients with moderate–severe ARDS, if protective ventilation strategies are applied.

Understanding the pathophysiological mechanism of barotrauma in covid patients is useful for the development of preventive interventions.

## Figures and Tables

**Table 1:** The baseline characteristics of 497 COVID-19 patients who underwent PPV.

	PNX/PNM Group (*n* = 36)	Non-PNX/PNM Group (*n* = 461)	*p*-Value ^a^
Age, median (IQR)	61.5 (55.7–71.5)	65 (57–74)	0.280
Male, *n* (%)	29 (80.5%)	340 (74%)	
Female, *n* (%)	7 (19.4%)	121 (26%)	
BMI, kg/m^2^, median (IQR)	26.9 (26–29.4)	27.8 (25.4–31.1)	0.286
SOFA score *, median (IQR)	5.5 (3–7.7)	4 (2–7)	0.209
APACHE II score *, median (IQR)	11 (8–18)	9 (7–16)	0.394
Comorbidities, *n* (%)			
Arterial hypertension	23 (64%)	248 (53.8%)	0.423
Other cardiopathies	7 (19.4%)	106 (23%)	0.949
Diabetes	5 (13.8%)	74 (16%)	0.271
Kidney disease (stage 3–5 of CKD)	1 (2.8%)	21 (4.5%)	0.438
Moderate to severe chronic liver disease	0 (0.0%)	5 (1.1%)	0.485
Chronic pulmonary diseases **	6 (16.7%)	58 (12.5%)	0.582
Neoplasm ***	3 (8.3%)	29 (6.3%)	0.974
Previous surgery in last month	2 (5.5%)	12 (2.6%)	0.396
Previous hospitalization in last six months	3 (8.3%)	24 (5.2%)	0.932
Obesity ****	11 (30.5%)	163 (35.4%)	0.147
Chronic neurological disorders	0 (0.0%)	38 (8.2%)	0.446
Autoimmune disease	3 (8.3%)	49 (10.7%)	0.508
Other chronic disease	7 (19.4%)	61 (13.2%)	0.436
Outcome at 30 days from barotrauma			
Discharged, patients (%)	19 (52.8%)	289 (62.6%)	
Mortality, patients (%)	17 (47.2%)	172 (37.3%)	0.429

Abbreviations: IQR, interquartile range; PNX, pneumothorax; PNM, pneumomediastinum; BMI, body mass index; SOFA score, sequential organ failure assessment score; APACHE II score, acute physiology and chronic health evaluation. * At hospital admission; ** include COPD, emphysema bullae or bronchial asthma; *** neoplasm in the last 5 years (solid neoplasia/hematological malignancy); **** obesity is defined as BMI > 30 kg/m^2^; CKD, stages of chronic kidney disease; ^a^ chi-square test was performed between the two groups.

## Data Availability

The data presented in this study are available on request from the corresponding author. The data are not publicly available because of patient privacy and data protection regulations.
